# Impact of the COVID-19 Pandemic on Drug-Resistant Tuberculosis in Europe: A Meta-Analysis of Epidemiological Trends

**DOI:** 10.3390/ph18101535

**Published:** 2025-10-12

**Authors:** Christina Zouganeli, Dimitra K. Toubanaki, Ourania Karaoulani, Georgia Vrioni, Evdokia Karagouni, Antonia Efstathiou

**Affiliations:** 1 Department of Biomedical Sciences, University of West Attica, 12243 Aigaleo, Greece; zouganelichristina24@gmail.com; 2 Immunology of Infection Group, Department of Microbiology, Hellenic Pasteur Institute, 11521 Athens, Greece; dtouban@pasteur.gr (D.K.T.); ekaragouni@pasteur.gr (E.K.); 3 National Reference Laboratory for Bovine Tuberculosis, Directorate of Veterinary Centre of Athens, Ministry of Rural Development and Food, 15310 Athens, Greece; okaraoulani@minagric.gr; 4 Department of Microbiology, Medical School, National and Kapodistrian University of Athens, 11527 Athens, Greece; gvrioni@med.uoa.gr

**Keywords:** tuberculosis, *Mycobacterium tuberculosis*, meta-analysis, European Union, ECDC, COVID-19, pandemic, development of resistance, antimicrobial resistance

## Abstract

**Background/Objectives**: The COVID-19 pandemic has significantly intensified global concerns surrounding antimicrobial resistance (AMR), particularly in relation to tuberculosis (TB). In the European Union (EU), the reallocation of healthcare resources towards managing COVID-19 led to a de-prioritization of TB surveillance and control. This shift contributed to delays in TB diagnosis and treatment, creating conditions favorable for the emergence and spread of drug-resistant TB strains. This meta-analysis aims to assess epidemiological trends of drug-resistant TB across EU countries before, during, and after the pandemic and quantify the impact of COVID-19 on *Mycobacterium tuberculosis* resistance patterns. **Methods:** Data were obtained from the European Centre for Disease Prevention and Control (ECDC) covering 2015 to 2022. Following the TB incidence, the multidrug-resistant TB (MDR-TB) and rifampicin-resistant/MDR-TB (RR/MDR-TB) cases, as well as treatment success rates over 12- and 24-month periods, were analyzed. The analysis included 31 EU countries across three-time frames: pre-pandemic (2015–2019), pandemic onset (2020), and post-pandemic transition (2020–2022). **Results**: The pandemic was associated with a decrease in reported TB cases but a simultaneous increase in the proportion of MDR and RR/MDR cases. Treatment success rates showed a modest rise for 24-month regimens, while outcomes declined for 12-month therapies. **Conclusions:** These findings underscore the pandemic’s disruptive impact on TB control and highlight the need for renewed investment in diagnostic capacity, treatment access, and antimicrobial stewardship, in order to reduce antimicrobial resistance occurrence. Continued monitoring beyond 2022 is essential to fully understand long-term effects and inform future public health strategies.

## 1. Introduction

Tuberculosis (TB) is a highly contagious disease with significant transmissibility and mortality [1]. Specifically, TB is a potentially fatal airborne infection, and its primary causative agent is *Mycobacterium tuberculosis* [2]. *M. tuberculosis* belongs to the *Mycobacterium* genus, which consists of aerobic, non-motile, Gram-positive, acid-fast bacilli bacteria species that are incapable of forming spores. *Mycobacterium tuberculosis* is distinguished by its slow growth rate and the intricate composition of its cell wall, factors that contribute to its heightened resistance to various antibiotics and therapeutic interventions relative to other microorganisms [3]. In general, mycobacteria are divided into two major groups: the MTBC (*Mycobacterium Tuberculosis* Complex) and the NTM (Non-Tuberculous *Mycobacteria*) [4]. Members of the MTBC can cause TB in animals and humans, while the NTM species cause opportunistic infections, particularly in immunocompromised individuals [4,5]. TB remains a major global health issue, with millions of cases reported annually. It is estimated that in 2020, tuberculosis affected approximately 10 million individuals and was responsible for around 1.5 million deaths, underscoring its status as a persistent global health threat with the potential to escalate in the coming years. For the first time in several years, a decline in the notification of newly diagnosed tuberculosis cases has been observed, concurrently with an increase in tuberculosis-related mortality. These trends are likely attributable to the disruptions in healthcare services and disease surveillance associated with the COVID-19 pandemic [1].

In humans, tuberculosis is transmitted when aerosolized droplets containing the pathogen, expelled through coughing or sneezing by an infected individual, are inhaled by a susceptible person, allowing the bacteria to enter the respiratory system via the upper respiratory tract. *Mycobacterium tuberculosis* can cause two forms of tuberculosis: pulmonary and extrapulmonary. The pulmonary form represents the most prevalent manifestation of the disease and serves as its primary mode of transmission [6]. The main symptoms of pulmonary tuberculosis include chronic productive cough, low-grade fever, hemoptysis, loss of appetite, night sweats, fatigue, weight loss, and general discomfort [7]. However, some patients may exhibit the extrapulmonary form of the disease, which includes symptoms depending on the organs affected [8,9]. Extrapulmonary tuberculosis may manifest as lymphadenitis, meningitis, disseminated miliary disease, or with symptoms involving the kidneys, bones, or joints. Diagnosis is established through a combination of clinical evaluation of the patient’s symptoms, laboratory investigations, and imaging examinations.

The standard treatment for tuberculosis typically involves a combination of first-line antimicrobial agents. Initially, a two-month intensive phase is administered, comprising rifampin, ethambutol, isoniazid, and pyrazinamide. This is followed by a continuation phase lasting four months, during which rifampin and isoniazid are prescribed [10,11]. Thus, the total treatment duration of this therapeutic scheme is approximately six months. If the *Mycobacterium tuberculosis* strain infecting the host is sensitive to first-line antibiotics and the patient strictly adheres to the prescribed medication protocol, the treatment is generally successful. However, if either of these conditions is not met, a longer treatment period is required. Consequently, in cases of multidrug-resistant tuberculosis (MDR-TB), treatment necessitates the use of second-line agents, such as fluoroquinolones, which often results in extended treatment durations and a heightened risk of adverse effects [6].

Over time, the emergence and escalation of bacterial resistance to antibiotics have become a significant public health concern. Antimicrobial resistance (AMR) compromises the efficacy of existing antimicrobial therapies, contributing to increased morbidity and mortality. AMR primarily arises from genetic mutations within bacterial populations and is further intensified by the inappropriate and excessive use of antibiotics. Regarding *Mycobacterium tuberculosis*’ resistance, there has been a concerning rise in the incidence of tuberculosis, particularly with respect to multidrug-resistant TB (MDR-TB) and extensively drug-resistant TB (XDR-TB), highlighting a critical challenge for global tuberculosis control efforts TB [12,13]. Multidrug-resistant tuberculosis (MDR-TB) is caused by strains of *Mycobacterium tuberculosis* that are resistant to at least isoniazid and rifampin, the two most potent first-line anti-tuberculosis drugs. Extensively drug-resistant tuberculosis (XDR-TB) represents a more severe form of resistance, characterized by additional resistance to any fluoroquinolone and at least one second-line injectable agent, such as amikacin, kanamycin, or capreomycin. Both MDR-TB and XDR-TB pose substantial challenges to tuberculosis control due to limited therapeutic options, prolonged treatment durations, and poorer clinical outcomes.

The COVID-19 pandemic has substantially intensified the global burden of antimicrobial resistance, including the emergence and escalation of AMR in tuberculosis. Disruptions to healthcare systems during the pandemic adversely affected TB diagnosis, treatment adherence and antibiotic stewardship. In the European Union (EU), healthcare resources were predominantly reallocated to address COVID-19, leading to the de-prioritization of TB surveillance and control efforts. This shift contributed to delays in TB detection and treatment, potentially increasing the incidence of drug-resistant TB cases. Moreover, the WHO reported widespread overuse of antibiotics among hospitalized COVID-19 patients, despite a low prevalence of bacterial co-infections. Between 2020 and 2022, approximately 75% of COVID-19 patients received antibiotic therapy, although only 8% had confirmed bacterial infections. This indiscriminate use of antibiotics likely accelerated the development and spread of AMR [14].

In fact, in the EU/EEA (European Economic Area), while there was a reported decrease in overall antibiotic consumption during the pandemic, the AMR levels remained high for several critical bacterial species and antibiotic combinations. The ECDC highlighted that the health burden of antibiotic-resistant infections in the region is comparable to that of influenza, tuberculosis, and HIV/AIDS combined, underscoring the severity of the AMR threat [15,16,17]. All mentioned facts emphasize the urgent need for strengthened antimicrobial stewardship, sustained investment in TB control programs, and comprehensive strategies to mitigate the impact of AMR in the post-pandemic era.

The present meta-analysis study seeks to investigate the epidemiological trends of treatment-resistant tuberculosis cases across Europe in the periods preceding and during the COVID-19 pandemic. The primary objective is to assess whether the pandemic had an impact on the resistance patterns of *Mycobacterium tuberculosis*, and, if so, to quantify the extent of this influence.

## 2. Materials and Methods

The meta-analysis covered the countries of the European Union, which in 2015–2022 reported on the number of total reported cases of TB, MDR-TB cases, RR/MDR-TB cases, treatment success for MDR-TB cases 24-month and 12-month therapies to the European Center for Disease Prevention and Control (ECDC). In total, 31 countries were analyzed: Austria, Belgium, Bulgaria, Croatia, Cyprus, Czechia, Denmark, Estonia, Finland, Germany, Greece, Hungary, Iceland, Ireland, Italia, Latvia, Liechtenstein, Lithuania, Sweden, Luxembourg, Malta, the Netherlands, Norway, Poland, Portugal, Romania, Slovakia, Slovenia, Spain and UK.

### 2.1. Protocol and Registration

This systematic review was reported according to the Preferred Reporting Items for Systematic Reviews and Meta-Analyses (PRISMA) guidelines (Supplementary Materials: Prisma checklist) and was prospectively registered on PROSPERO (CRD420251153770, https://www.crd.york.ac.uk/PROSPERO/view/CRD420251153770, accessed on 25 September 2025).

### 2.2. Data

Theoretical data were collected through an extensive literature review via the PubMed database. These data were used to support the theoretical framework and analyze key concepts of the meta-analysis.

Data on the number of total reported cases of TB, MDR-TB cases, RR/MDR-TB cases, treatment success rates for MDR-TB cases undergoing 24-month and 12-month therapies were obtained from the European Center for Disease Prevention and Control (ECDC) website: https://atlas.ecdc.europa.eu/public/index.aspx (accessed on 1 July 2025). The data from 2015 to 2022, were used to examine the impact of the COVID-19 pandemic on the epidemiological spread of treatment-resistant TB cases in the European Union and its members individually.

Three periods were analyzed: the pre-pandemic period (2015–2019), the COVID-19 pandemic year (2020) and post-pandemic period (2020–2022).

### 2.3. Statistical Analysis

The graphical analysis of the data provided was conducted using the statistical software GraphPad Prism (version 8.01), covering the period from 2015 to 2022, up to the most recently published data. Thus, all figures were generated by our team using Prism software based on official ECDC epidemiological data which have not been previously published and contain no duplications or inconsistencies.

## 3. Results

**Figure 1:**
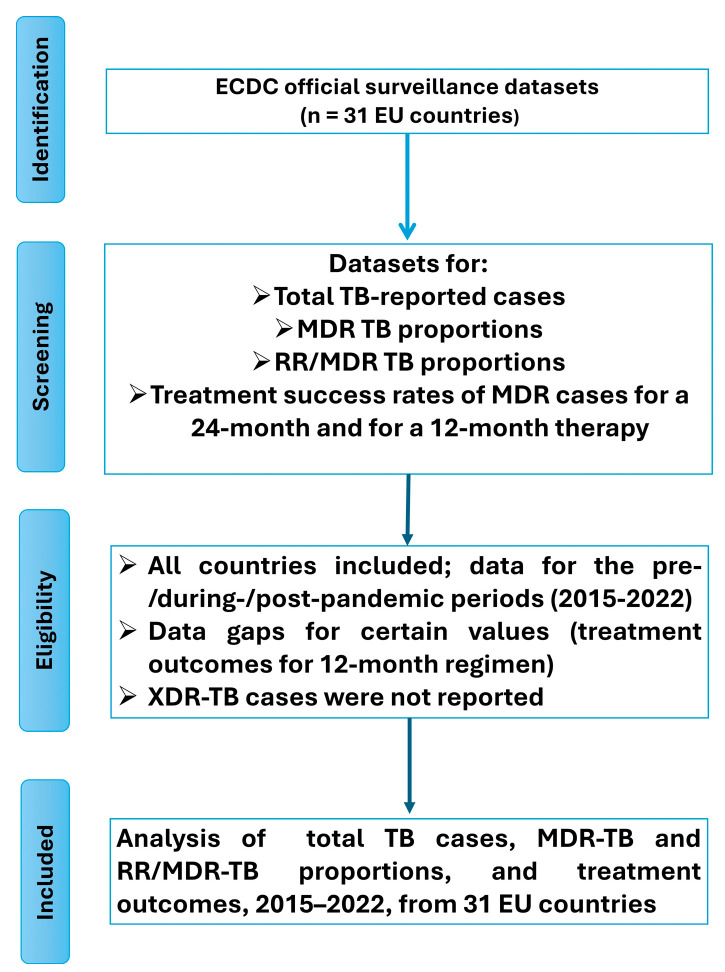
PRISMA study flow diagram.

This meta-analysis study evaluates the total number of reported tuberculosis cases, the proportion of multidrug-resistant (MDR) cases, the proportion of rifampicin-resistant/multidrug-resistant (RR/MDR) cases, and the rates of successful treatment outcomes at 12 and 24 months. These data are stratified by country and year. The results are presented with an emphasis on temporal comparisons, specifically between the pre-pandemic period (2015–2019), the onset of the pandemic (2019–2020), and the pandemic period (2020–2022). The data presentation is followed by critical analysis and interpretation of the findings. All data used for the present analysis were obtained from official reports published by the European Centre for Disease Prevention and Control (ECDC). The data selection process is illustrated in Figure 1.

### 3.1. Total TB-Reported Cases

Between 2015 and 2019, the total number of reported tuberculosis cases in the European Union declined from 54,721 to 45,201, representing a 17.39% reduction. However, during the COVID-19 pandemic, a marked decline in reported TB cases was observed, largely attributable to the widespread implementation of quarantine measures and movement restrictions across EU member states. In 2020, the number of recorded TB cases dropped further to 33,805, reflecting a 25% decrease compared to the previous year. This substantial reduction is believed to primarily result from underreporting and decreased case detection during the pandemic, a pattern similarly observed for other non-COVID-19-related diseases. In 2021, the number of reported tuberculosis cases in the European Union remained relatively stable. However, following the relaxation of quarantine and mobility restrictions, an increase in case notifications was observed, with 36,179 cases reported in 2022—an increase of 7.44% compared to the 33,676 cases recorded in 2021. Despite this rise, the number of cases in 2022 remained 19.96% lower than in 2019, the year preceding the onset of the COVID-19 pandemic. This overall decline is likely attributable to a combination of factors, including underreporting during the pandemic and the impact of public health measures introduced to mitigate the spread of SARS-CoV-2, which concurrently reduced the transmission of other infectious diseases such as tuberculosis.

**Figure 2:**
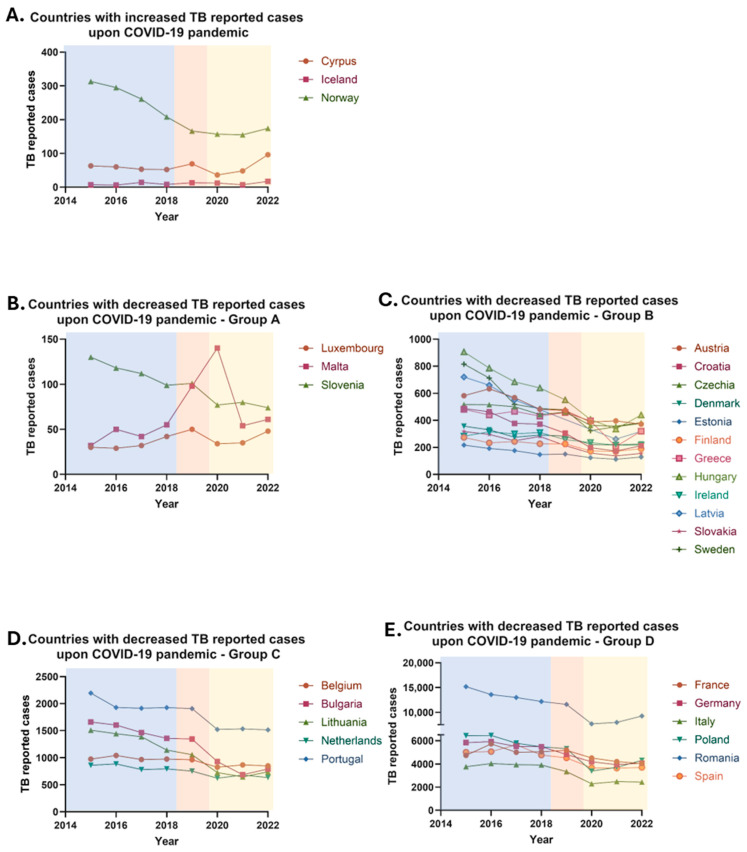
Tuberculosis (TB) annually reported cases in the European Union/European Economic area (EU/EEA) from 2015 to 2022. (**A**) Countries with increased TB reported cases upon COVID-19 pandemic. (**B**) Countries with decreased TB reported cases upon COVID-19 pandemic (group A). (**C**) Countries with decreased TB reported cases upon COVID-19 pandemic (group B). (**D**) Countries with decreased TB reported cases upon COVID-19 pandemic (group C). (**E**) Countries with decreased TB reported cases upon COVID-19 pandemic (group D).

Specifically, from 2019 to 2022, a decline in the total number of tuberculosis cases was observed in 26 countries of the European Union, while an increase was recorded in three EU countries, and one country showed a stable case count (Figure 2). The increase in cases was highest in Cyprus (39.13%) and in Iceland (30.77%). Norway on the other hand experienced a much smaller rise (4.82%) (Figure 2A). Notably, a decrease was noted in 83.87% of EU countries, including Austria, Belgium, Bulgaria, Croatia, the Czech Republic, Denmark, Estonia, Finland, France, Germany, Greece, Hungary, Ireland, Italy, Latvia, Lithuania, Sweden, Luxembourg, Malta, the Netherlands, Poland, Portugal, Romania, Slovakia, Slovenia, and Spain (Figure 2B–E). It is important to note that the greatest reduction in cases was observed in Bulgaria (41.07%), Latvia (42.21%), and Malta (37.76%). Conversely, an increase was observed in 9.68% of EU countries, specifically in Cyprus, Iceland, and Norway. Lastly, the number of cases remained stable in Liechtenstein (3.23% of EU countries) compared to pre-pandemic data. No comparison was made for the UK due to lack of post-2019 data (Table 1).

### 3.2. Multidrug-Resistant (MDR) and Rifampicin-Resistant/Multidrug-Resistant (RR/MDR) Cases

#### 3.2.1. MDR Cases

Between 2015 and 2019, the proportion of multidrug-resistant (MDR) tuberculosis cases in the European Union (EU) declined from 4.4% to 3.2% of the total reported TB cases. In 2020, however, the percentage of MDR cases slightly increased to 3.3%.

During the COVID-19 pandemic (2020–2021), the proportion of reported MDR cases remained relatively stable, likely due to the widespread quarantine measures implemented across most EU countries. Following the relaxation of these restrictions, an increase in MDR cases was observed in 2022, with the proportion rising from 3.3% to 4.0% of total reported TB cases. Overall, comparing the pre-pandemic period (2019), when 3.2% of reported TB cases identified as MDR, with the post-pandemic period (2022), when 4% were MDR, shows an approximate 25% relative increase in the proportion of MDR cases in the EU.

**Figure 3:**
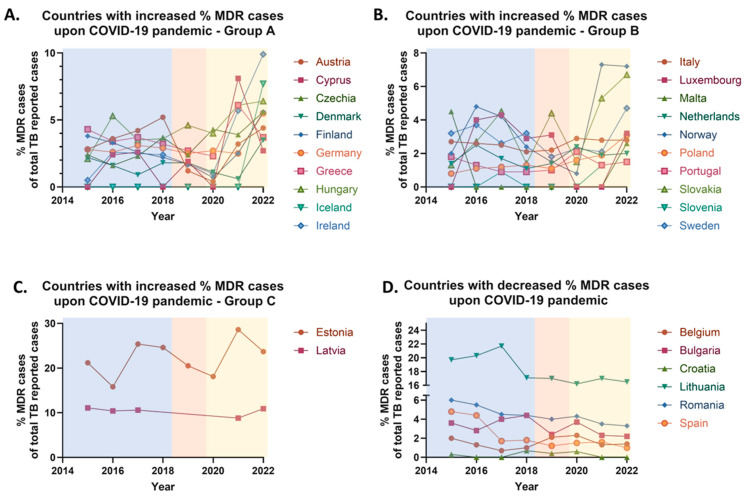
Percentage rates of multidrug-resistant (MDR) tuberculosis (TB) annually reported cases in the European Union/European Economic area (EU/EEA) from 2015 to 2022. (**A**) Countries with increased % MDR reported cases upon COVID-19 pandemic (group A). (**B**) Countries with increased % MDR reported cases upon COVID-19 pandemic (group B). (**C**) Countries with increased % MDR reported cases upon COVID-19 pandemic (group C). (**D**) Countries with decreased % MDR reported cases upon COVID-19 pandemic.

Between 2019 and 2022, an increase in the proportion of multidrug-resistant (MDR) tuberculosis cases was observed in 22 European Union (EU) countries with an average of 154% increase in MDR cases’ rate, while a decrease was reported in 6 countries (average decrease ≈ 30% of MDR cases’ rate); no country reported a stable MDR rate over this period. Notably, 70.97% of EU countries—including Austria, Cyprus, Czechia, Denmark, Estonia, Finland, Germany, Greece, Hungary, Iceland, Ireland, Italy, Latvia, Luxembourg, Malta, the Netherlands, Norway, Poland, Portugal, Slovakia, Slovenia, and Sweden—recorded an increase in the percentage of MDR cases (Figure 3A–C). Conversely, a decrease was observed in 19.35% of countries, specifically Belgium, Bulgaria, Croatia, Lithuania, Romania, and Spain (Figure 3D). Comparative data for France, Liechtenstein, and the UK were unavailable due to the absence of post-2019 reporting (Table 2).

#### 3.2.2. RR/MDR Cases

Between 2015 and 2019, the proportion of rifampicin-resistant/multidrug-resistant (RR/MDR) tuberculosis cases in the European Union (EU) declined from 4.6% to 3.7% of the total TB reported cases. In 2020, however, the percentage of RR/MDR cases increased to 3.9% of the total TB reported cases. During the COVID-19 pandemic (2020–2021), the reported proportion of RR/MDR cases remained relatively stable, likely due to the widespread implementation of quarantine measures across most EU countries. Following the relaxation of these restrictions, an increase was observed in 2022, with the RR/MDR proportion rising from 3.9% to 4.6% of the total TB reported cases. Overall, between the pre-pandemic year 2019 and the post-pandemic year 2022, the percentage of RR/MDR cases in the EU increased by 24.32%.

**Figure 4:**
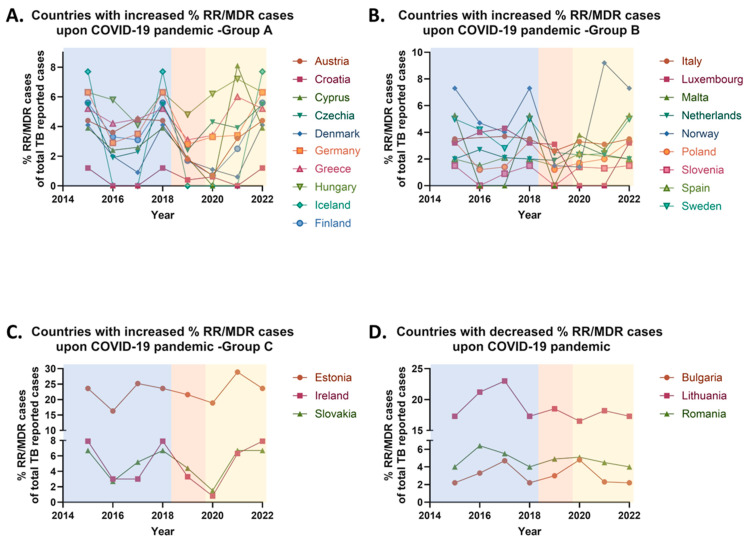
Percentage rates of rifampicin-resistant/multidrug-resistant (RR/MDR) tuberculosis (TB) annually reported cases in the European Union/European Economic area (EU/EEA) from 2015 to 2022. (**A**) Countries with increased % RR/MDR reported cases upon COVID-19 pandemic (group A). (**B**) Countries with increased % RR/MDR reported cases upon COVID-19 pandemic (group B). (**C**) Countries with increased % RR/MDR reported cases upon COVID-19 pandemic (group C). (**D**) Countries with decreased % RR/MDR reported cases upon COVID-19 pandemic.

More specifically, between 2019 and 2022, an increase in the proportion of RR/MDR tuberculosis cases, was observed in 22 European Union (EU) countries with an average of 162% increase in RR/MDR cases’ rate, while a decrease was reported in 3 countries (average decrease ≈ 17% of RR/MDR cases’ rate), and 3 countries exhibited stable RR/MDR rates. Specifically, 70.97% of EU countries—including Austria, Croatia, Cyprus, Czechia, Denmark, Estonia, Finland, Germany, Greece, Hungary, Iceland, Ireland, Italy, Luxembourg, Malta, the Netherlands, Norway, Poland, Slovakia, Slovenia, Spain, and Sweden—reported an increase (Figure 4A–C), while 9.68% of countries, namely Bulgaria, Lithuania, and Romania, recorded a decrease in their RR/MDR rates (Figure 4D). A stable proportion of RR/MDR cases was noted in Belgium, Latvia, and Portugal, also representing 9.68% of the EU countries. No comparative analysis was conducted for France, Liechtenstein, and the UK due to the absence of data after 2019 (Table 3).

Between 2019 and 2022, the most significant increases in the proportions of multidrug-resistant and rifampicin-resistant/multidrug-resistant tuberculosis cases were recorded in eight European Union countries, representing 25.8% of all EU member states. In each of these countries, the rise exceeded 100%. The countries and their respective increases were as follows: Austria, Czechia, Finland, Germany, Ireland, Norway, Poland and Sweden (Table 4).

Ιt is evident that the majority of countries exhibiting the most pronounced increases are situated in Central and Northern Europe. Notably, the highest increase in both MDR and RR/MDR tuberculosis cases were observed in Norway. Moreover, Denmark demonstrated a substantial 200% increase in RR/MDR cases and a notable 94.44% rise in MDR cases, but it was not included among the top eight countries, since the increase in MDR cases did not exceed 100%. Similarly, Croatia, despite a 200% rise in RR/MDR cases, was excluded due to a concurrent complete (100%) decrease in MDR cases.

It is important to emphasize that the trends in the proportions of MDR and RR/MDR tuberculosis cases were not uniform across all EU countries. A divergence between the percentages of MDR and RR/MDR cases was observed in five member states—specifically Belgium, Croatia, Latvia, Portugal, and Spain (Table 2, Table 3 and Table 5). The potential factors contributing to these discrepancies will be explored in the subsequent sections.

### 3.3. Treatment Success Rates of MDR Cases

Monitoring treatment success rates in multidrug-resistant (MDR) tuberculosis cases is essential for evaluating the effectiveness of public health interventions and guiding therapeutic strategies, particularly in the context of external disruptions such as the COVID-19 pandemic. The ECDC provides annual data on treatment outcomes, offering valuable insights into temporal trends across the European Union. Given the significant impact of the pandemic on healthcare systems, this section aims to analyze changes in treatment success rates at both 12- and 24-month follow-up points. Specifically, data on 24-month treatment success rates for MDR tuberculosis cases, as reported on the ECDC website, were available for the period from 2015 to 2020. Aim of the analysis was to compare the 24-month treatment success rates before and during the COVID-19 pandemic, specifically between 2019 and 2020, given the absence of officially reported post-pandemic data (2021–2022). Similarly, data on 12-month treatment success rates for MDR cases were available from 2015 to 2021 and the present analysis compares the 12-month treatment outcomes before and during the pandemic, focusing on the years 2019 and 2021, due to the unavailability of officially reported data beyond 2022.

#### 3.3.1. Treatment Success Rates of MDR Cases for a 24-Month Therapy

Between 2015 and 2019, the 24-month treatment success rates for multidrug-resistant tuberculosis in the European Union increased from 43.9% to 47.9%, reflecting a 9.11% rise in the treatment success. In 2020, following the onset of the COVID-19 pandemic, the treatment success rates further increased to 48.6%, likely attributed to the quarantine restrictions implemented across most EU countries. However, after the relaxation of these restrictions, no officially reported data on the 24-month treatment success rates for MDR cases have been made available.

**Figure 5:**
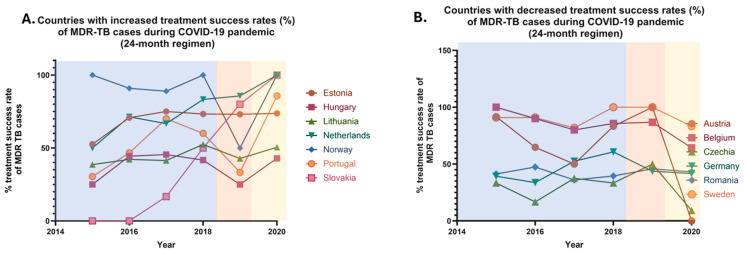
Percentage rates of treatment success of multidrug resistant (MDR) tuberculosis (TB) annually reported cases in the European Union/European Economic area (EU/EEA) from 2015 to 2020, in a 24-month therapeutic regimen. (**A**) Countries with increased treatment success rates (%) of MDR TB cases during COVID-19 pandemic. (**B**) Countries with decreased treatment success rates (%) of MDR TB cases during COVID-19 pandemic.

Specifically, between 2019 and 2020, a rise in the 24-month treatment success rates was observed in seven EU countries, while a decline was reported in six countries (Figure 5). Stable rates were noted in only one country. Noteworthy increases were recorded in 22.58% of all EU countries, including Estonia, Hungary, Lithuania, the Netherlands, Norway, Portugal, and Slovakia. In contrast, a decrease was reported in 19.35% of all EU countries, including Austria, Belgium, the Czech Republic, Germany, Romania, and Sweden. Stable treatment success rates between the pre-pandemic and pandemic periods were only observed in Croatia. No comparisons were made for 17 EU countries, due to the absence of officially reported data. These countries—Bulgaria, Cyprus, Denmark, Finland, France, Greece, Iceland, Ireland, Italy, Latvia, Liechtenstein, Luxembourg, Malta, Poland, Slovenia, Spain, and the UK—represent 54.84% of the EU member states (Table 6).

#### 3.3.2. Treatment Success Rates of MDR Cases-12-Month Therapy

From 2015 to 2019, the 12-month treatment success rates for multidrug-resistant tuberculosis in the European Union increased from 62.9% to 65.4%, reflecting a 3.97% rise in treatment success. However, in 2020, following the onset of the COVID-19 pandemic, the 12-month treatment success rates decreased to 62.7%, likely due to the quarantine restrictions implemented across most EU countries. In 2021, the treatment success rates remained stable (62.9%). Overall, from 2019 to 2021, the 12-month treatment success rates for MDR cases declined by 3.82%. Following the easing of quarantine restrictions, no officially reported data on the 12-month treatment success rates for MDR cases has been available.

**Figure 6:**
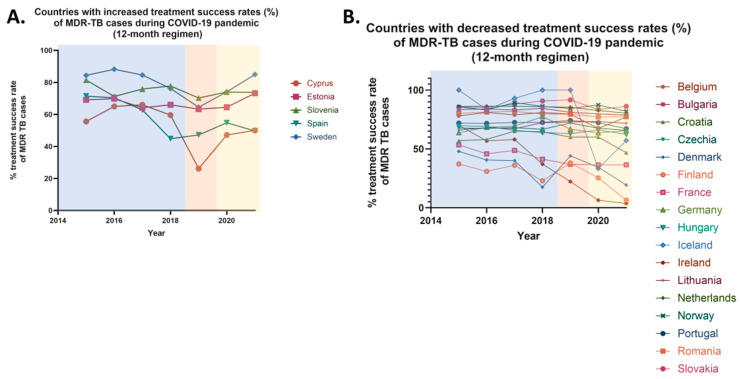
Percentage rates of treatment success of multidrug resistant (MDR) tuberculosis (TB) annually reported cases in the European Union/European Economic area (EU/EEA) from 2015 to 2021, in a 12-month therapeutic regimen. (**A**) Countries with increased treatment success rates (%) of MDR TB cases during COVID-19 pandemic. (**B**) Countries with decreased treatment success rates (%) of MDR TB cases during COVID-19 pandemic.

Specifically, between 2019 and 2021, an increase in 12-month treatment success rates was observed in 5 EU countries, while a decrease was reported in 17 countries (Figure 6). Stable rates were noted only in two countries. More specifically, increases were observed in 16.13% of EU countries, including Cyprus, Estonia, Slovenia, Spain, and Sweden. In contrast, a decrease was recorded in 54.84% of EU countries, including Belgium, Bulgaria, Croatia, Czechia, Denmark, Finland, Germany, Hungary, Iceland, Ireland, Lithuania, the Netherlands, Norway, Portugal, Romania, and Slovakia. Stable rates were noted in Austria and Liechtenstein when comparing pre-pandemic and pandemic periods. No comparison was made for seven countries due to the absence of officially reported data. These countries—Greece, Italy, Latvia, Luxembourg, Malta, Poland, and the UK—represent 22.58% of the EU member states (Table 7).

It is noteworthy that significant variations exist between the treatment success rates for 24-month and 12-month therapies for multidrug-resistant (MDR) tuberculosis across European Union (EU) countries. These variations were observed in nine countries, which collectively represent 29.03% of the EU member states, including Austria, Croatia, Hungary, Lithuania, Sweden, the Netherlands, Norway, Portugal, and Slovakia. In Austria, a decrease in the treatment success rate for 24-month therapy was reported (declining from 100% in 2019 to 0% in 2020), while the success rate for 12-month therapy remained stable. In contrast, Croatia exhibited a stable 24-month therapy success rate, but a decline was noted for the 12-month therapy (declining from 60% in 2019 to 46.8% in 2021). Sweden is the only country where a reduction in the 24-month therapy success rate (declining from 100% in 2019 to 83.3% in 2020), was accompanied by an increase in the 12-month therapy success rate (increasing from 64.5% in 2019 to 85% in 2021). In the remaining six countries, an increase in the 24-month treatment success rate was observed (average increase ≈65%), alongside a decrease in the 12-month therapy success rate (average decrease ≈4.5%). Additionally, Estonia stands out as the sole country where both treatment success rates increased, with a slight rise of 0.82% in the 24-month therapy and a more substantial increase of 15.64% in the 12-month therapy for MDR cases. A simultaneous decline in both the 24-month and 12-month therapy success rates was reported in four countries, representing 12.90% of the EU: Austria, Belgium, Czechia, Germany and Romania (Table 8).

Finally, the absence of officially reported data, particularly regarding 24-month therapy outcomes, presents a limitation in fully assessing the extent to which variations in treatment success rates exist across the EU. This data gap may hinder a comprehensive evaluation of the true extent of these variations.

## 4. Discussion

According to official data from the European Centre for Disease Prevention and Control (ECDC), the COVID-19 pandemic has had a significant impact on tuberculosis (TB) trends across the European Union (EU). While the overall decline in TB cases during the pandemic may appear encouraging and aligns with the World Health Organization’s (WHO) goal of TB elimination by 2030 [18,19], these figures must be interpreted cautiously. In general, it should be noted that the observed changes in TB incidence and drug-resistance patterns cannot be attributed solely to the COVID-19 pandemic, as other factors—such as changes in healthcare access, mortality from competing diseases, and broader social determinants—likely contributed to these trends, which are further addressed in the Section 4.

The observed reduction in TB cases across most EU countries is multifactorial. Primarily, it reflects decreased access to healthcare services and the overall strain on health systems during the pandemic, leading to under-diagnosis and underreporting of TB cases [19]. Additionally, non-pharmaceutical interventions such as social distancing, lockdowns, mask usage, and reduced mobility—implemented to control the spread of COVID-19—may have inadvertently suppressed TB transmission, as both diseases share similar routes of airborne transmission. Notable declines in TB incidence were reported in Bulgaria (−41.07%), Latvia (−42.21%), and Malta (−37.76%), suggesting that these countries were particularly affected by diagnostic disruptions or benefited more from preventive measures.

Conversely, a few EU countries—including Cyprus (+39.13%), Iceland (+30.77%), and Norway (+4.82%)—reported increases in TB notifications. These trends may reflect delayed diagnoses due to healthcare service disruptions during the pandemic, with a subsequent rebound once restrictions were lifted. In some cases, reactivation of latent TB or treatment interruptions during the pandemic may also have contributed. Migration may represent an additional factor, particularly in countries like Cyprus, Ireland, and Norway, which are known destinations for asylum seekers and refugees—populations at increased risk of TB. Liechtenstein presented a unique case, maintaining a stable TB incidence with one case reported in both 2018 and 2022. This consistency is likely attributable to the country’s small population size, limited migration, robust healthcare infrastructure, and favorable socio-economic conditions, which together facilitate effective TB control.

Despite a general decline in tuberculosis cases during the COVID-19 pandemic, an increase in the proportion of multidrug-resistant and rifampicin-resistant/multidrug-resistant TB cases, was observed in several European countries. This trend is likely associated with treatment interruptions and non-adherence due to restricted healthcare access, medication shortages, and delays in diagnosis. Inadequate drug dosages and insufficient patient monitoring may have contributed to prolonged bacterial survival, promoting resistance development. Additionally, inappropriate antibiotic use during the pandemic likely facilitated the emergence of resistant *Mycobacterium tuberculosis* strains [19].

Conversely, a decrease in MDR and RR/MDR TB cases was reported in Bulgaria, Romania, and Lithuania. This decline may reflect improved treatment adherence, increased access to modern therapies, and more effective implementation of World Health Organization guidelines. The Directly Observed Treatment Short-course (DOTS) strategy, which ensures compliance with treatment regimens, has likely played a key role in these improvements [20]. Enhanced public awareness and education may have also contributed. However, the potential influence of underreporting or reduced diagnostic capacity during the pandemic must be considered. Romania, which has historically had the highest TB burden in the EU, shows signs of improved drug-resistant TB management, likely due to advancements in diagnostics and greater adherence to WHO protocols. In Belgium, Latvia, and Portugal, the proportion of RR/MDR TB cases has remained stable. This may reflect robust healthcare systems, effective antibiotic stewardship, and containment of transmission sources. Alternatively, this stability may result from underreporting or data limitations.

It should be noted here that countries such as Belgium, Croatia, Latvia, Portugal, and Spain appear to have discrepancies between MDR and RR/MDR TB case rates. These variations may result from differences in diagnostic methodologies. Nations employing more sensitive molecular diagnostics (e.g., Xpert MTB/RIF) may detect rifampicin resistance more reliably, while others using culture-based diagnostics may identify a broader resistance spectrum. Regional differences in drug usage and monitoring practices may also influence resistance patterns. For instance, in settings with widespread rifampicin use and inadequate patient monitoring, increased RR/MDR rates may occur independently of MDR rates.

Improvements in treatment success rates over 12- and 24-month periods may be linked to enhanced healthcare services, particularly in diagnostics and treatment initiation. Implementation of monitoring strategies such as the Directly Observed Treatment (DOT) program ensures adherence to protocols [20]. Greater access to and timely administration of second-line therapies has likely contributed to better outcomes [2]. Notably, Estonia reported increases in both treatment success metrics: 0.82% for 24-month treatment and 15.64% for 12-month treatment. Conversely, some countries experienced declines in treatment effectiveness, likely due to COVID-19-related healthcare disruptions. Quarantine measures, resource shortages, and overwhelmed health systems contributed to diagnostic delays and treatment interruptions [19]. Inadequate treatment monitoring and premature discontinuation due to side effects or financial hardship may have also played roles [2]. These factors not only reduced treatment success but may have further promoted the emergence of drug-resistant strains.

Variations in treatment success between 12- and 24-month durations may stem from differences in national treatment protocols. Countries using modern therapies such as bedaquiline often report higher short-term (12-month) success but may face challenges in sustaining effectiveness over longer durations due to adherence issues. Conversely, nations emphasizing extended (24-month) regimens may show stronger long-term outcomes. Prolonged treatment courses, however, increase the risk of non-compliance due to side effects and economic barriers. Differences in drug availability and treatment strategies further explain observed discrepancies.

Countries maintaining stable proportions of treatment success over time may benefit from consistent healthcare infrastructure and strong TB control policies. Nonetheless, these stable figures may also reflect underreporting or limitations in data collection rather than actual improvements in treatment efficacy.

To improve tuberculosis prevention, novel integrated measures such as diagnosis and treatment that alleviate the drug-resistant tuberculosis global burden should be designed and implemented, especially since the COVID-19 pandemic seems to have caused an increase in the proportion of multidrug-resistant and rifampicin-resistant/multidrug-resistant TB cases in several European countries, as suggested by the present study. In order to provide a more complete perspective towards drug-resistant tuberculosis fighting, an overview of recent advances in the treatment of MDR, RR/MDR, and extensively drug-resistant (XDR) tuberculosis, both in clinical practice and ongoing research, is presented in Section 4.1

### 4.1. New Approaches Against Multidrug-Resistant (MDR), Rifampicin-Resistant (RR) and Extensively Drug-Resistant (XDR) Tuberculosis

The ongoing evolution of multidrug-resistant (MDR), rifampicin-resistant (RR), and extensively drug-resistant (XDR) tuberculosis has prompted the development of new and innovative approaches in both clinical practice and research. These advancements aim to enhance treatment regimens, improve diagnostic methods, and prioritize patient-centered care to effectively address the increasing global threat of drug-resistant TB. One of the most promising developments in tuberculosis treatment is the introduction of shorter, all-oral regimens consisting of novel drugs. These regimens combine newer medications like bedaquiline, pretomanid and linezolid, with or without moxifloxacin, depending on the resistance profiles of the bacteria [21,22,23]. For example, the 6-month BPaLM regimen (regimen constisting of bedaquiline, linezolid, pretomanid, and moxifloxacin) has shown improved efficacy and tolerability, providing a viable alternative to traditional longer regimens that often include toxic injectable drugs [14,21]. Additionally, for cases with fluoroquinolone resistance, modified versions such as the BPaL regimen are considered appropriate.

Significant progress has been made in diagnostics through the use of rapid molecular tests and whole-genome sequencing. Research into rapid molecular diagnostic tests has revolutionized the ability to detect drug-resistant TB early. Techniques like Xpert MTB/RIF and GeneXpert allow for rapid identification of resistance to key TB drugs, such as rifampicin, helping clinicians to tailor treatment more effectively [24,25]. Ongoing research aims to enhance these tools, making diagnostics faster, more portable, and accessible even in low-resource settings [24]. Innovations such as artificial intelligence are being integrated into radiology and genomic interpretation, which further improves diagnostic accuracy and clinical decision-making [26,27,28]. Moreover, whole-genome sequencing (WGS) offers an in-depth analysis of TB strains, enabling precise identification of genetic mutations associated with drug resistance. This method is being explored as a tool to improve diagnostic accuracy and track transmission patterns in real time, enhancing epidemiological surveillance and control measures [29,30].

New treatment models prioritize patient-centered care by integrating digital adherence tools, community health worker support, and telemedicine to tackle the challenges associated with long and complex TB treatment. In addition to clinical advancements, research is increasingly focused on social determinants of health. Factors such as poverty, stigma, and limited access to healthcare significantly impact treatment outcomes [31]. Interventions aimed at addressing these structural issues such as providing nutritional support, reducing stigma, and enhancing education are being implemented alongside medical care. Simultaneously, new research is focusing on understanding the social determinants of TB, such as poverty, crowded living conditions, and lack of access to healthcare, which contribute to the spread of drug-resistant TB. Interventions designed to address these factors, such as improving housing, nutrition, and education, are being tested as part of a comprehensive TB control strategy [32].

Public–private partnerships and global initiatives led by organizations like World Health Organization (WHO) and the Global Fund are playing a crucial role in the development and equitable distribution of new drugs, diagnostics, and care models. This combination of scientific innovation, patient-centered strategies, and international collaboration marks a significant shift in the way drug-resistant tuberculosis is being addressed today. The goal is not only to improve individual patient outcomes but also to hasten progress toward the elimination of TB [33].

#### 4.1.1. Treatment

Different forms of drug-resistant TB require different treatment regimens based on the type of resistance. According to WHO [14], drug-resistant TB is classified into five main categories: (i) isoniazid-resistant TB, (ii) RR-TB (rifampicin-resistant TB), (iii) MDR-TB (multidrug-resistant TB), (iv) pre-extensively drug-resistant TB (pre-XDR-TB), defined as TB that is resistant to rifampicin and any fluoroquinolone (a class of second-line anti-TB drug) and (v) XDR-TB, defined as TB that is resistant to rifampicin, plus any fluoroquinolone, plus at least one of either bedaquiline or linezolid [14].

Today, drug susceptible TB (DS-TB) is being treated with the ‘short-course chemotherapy’ regimen. This regimen consists of two phases. Initially, a two-month intensive phase is administered, comprising rifampin, ethambutol, isoniazid, and pyrazinamide. This is followed by a continuation phase lasting four months, during which rifampin and isoniazid are prescribed [10,11]. Thus, the total treatment duration of this therapeutic scheme is approximately six months. This regimen was believed to cure most patients from the first trials that took place between 1946 and 1986 by the British Medical Research council [34]. Later, pyrazinamide was added to the regimen in order to shorten the treatment from 9 to 6 months. As it became the global standard, it was used for treating all forms of DS-TB and undoubtedly it has saved millions of lives. Because of its lengthy duration many patients struggle to complete their treatment and there has been much interest in shortening the regimen. Despite its success in treating TB, the rise in drug-resistant cases highlights the urgent need for new treatment approaches.

As of 6 July 2023, there are some registered, unpublished clinical trials for the treatment of drug-susceptible tuberculosis which aim to either optimize the use of rifampicin or explore novel therapeutic approaches involving new drugs [21]. These clinical changes have demonstrated that rifampicin doses of up to 40 mg/kg/day are well tolerated associated with enhanced early bactericidal activity [35]. However, whether higher doses of rifampicin can safely reduce treatment duration or improve outcomes, in cases such as TB meningitis, this addition remains under investigation in the ongoing clinical trials [35]. Recent findings from a large phase III trial (Study 31/A5349), demonstrated that rifapentine, a rifamycin with an extended half-life, used in combination with isoniazid, pyrazinamide, and moxifloxacin, can reduce the duration of treatment for drug-susceptible TB to four months [36]. Based on these results, in May 2022, the WHO issued a conditional recommendation for the use of this 4-month regimen in eligible individuals aged 12 years and older with pulmonary DS-TB [37].

In response to the growing challenge of multidrug-resistant and rifampicin-resistant tuberculosis, several new pharmacological strategies have been developed. Historically, the management of MDR/RR-TB cases relied on prolonged treatment regimens, often extending up to 24 months, that included injectable agents (kanamycin and capreomycin), and which were associated with considerable toxicity and poor patient adherence. Recent advancements have led to the introduction of shorter, all-oral therapeutic regimens with improved safety (fewer side effects) and enhanced efficacy, as mentioned above (i.e., the BPaL regimen), showing promising results even in extensively drug-resistant TB cases. Clinical trials have demonstrated that these regimens yield higher treatment success rates while simultaneously improving patient adherence. Therefore, these new strategies constitute a crucial step forward in the global fight against TB [21].

The most recent WHO guidelines for drug-resistant TB treatment include three main types of treatment regimens [37,38]. The first category includes two all-oral treatment regimens lasting 6 months for people with MDR/RR-TB, regardless of whether resistance to fluoroquinolones is present [39]. The second category consists of several all-oral short regimens of approximately 9 months, suitable for patients with MDR/RR-TB who do not exhibit resistance to fluoroquinolones. The third category involves longer treatment regimens lasting 18 to 20 months, which may include an injectable drug such as amikacin. The 6-month regimens are prioritized as the preferred option, whereas the extended regimens are a last resort.

In line with WHO’s mission, all patients with MDR/RR-TB including those with additional resistance to fluoroquinolones, should have access to effective all-oral treatment regimens, whether they are shorter or longer, and implemented under programmatic conditions. In its 2022 update, the WHO drug-resistant TB treatment guidelines prioritized a 6-month regimen (BPaLM) as the preferred option for eligible patients. The 6-month BPaLM/BPaL regimen is composed of bedaquiline, pretomanid, linezolid (600 mg) and moxifloxacin. It can be used programmatically as a replacement for 9-month or longer (>18 months) regimens, in patients aged 14 years and older with MDR/RR-TB, provided they have not previously been exposed to bedaquiline, pretomanid, or linezolid (with exposure defined as >1 month). In case of documented resistance to fluoroquinolones, moxifloxacin may be omitted from this regimen (BPaL regimen). This is applicable to patients with pre-XDR-TB [38].

Additionally, the new 6-month BDLLfxC regimen can expand the use of shorter regimens to more patient groups, such as children, adolescents, and pregnant women, who have not been able to benefit from the currently recommended BPaLM regimen due to a lack of safety and dosing data for pretomanid. More specifically. The BDLLfxC regimen consists of bedaquiline, delamanid, linezolid (600 mg), levofloxacin, and clofazimine. It can be programmatically implemented as an alternative to 9-month or longer regimens (>18 months) in patients with MDR/RR-TB, provided there is no prior exposure (defined as >1 month) to bedaquiline, delamanid, or linezolid. Furthermore, in cases of fluoroquinolone resistance, this regimen may be adapted by omitting levofloxacin or clofazimine based on drug susceptibility testing (DST) results. Although DST for fluoroquinolones is strongly recommended, its availability should not delay the initiation of regimens that are still effective in pre-XDR-TB cases [38].

Apart from the 6-month regimens, there are 9-month all-oral regimens, which use is preferred over other currently recommended longer (18-month) regimens in patients with MDR/RR-TB, provided they have not previously been exposed to bedaquiline, delamanid and linezolid (with exposure defined as >1 month) and fluoroquinolones-resistance is not present. DST results are important for ruling out fluoroquinolone resistance and are required before the start of one of such regimens. Lastly, the 9-month all-oral regimens consisting of bedaquiline are preferred over the longer (>18 months) regimens children and adults with MDR/RR-TB, provided that there is no previous exposure to second-line treatment (including bedaquiline) and there is no resistance to fluoroquinolones. Simultaneously, there needs to be no extensive pulmonary TB disease or severe forms of extrapulmonary TB. Moreover, in these regimens, 2 months of linezolid can be implemented in place of 4 months of ethionamide [38].

Patients with advanced forms of drug-resistant TB, such as XDR-TB, or those who are ineligible for or have not responded to shorter treatment regimens, can benefit from individualized longer regimens (≥18 months). These regimens should be tailored based on the priority grouping of medications recommended in the most recent WHO guidelines [38].

Overall, decisions regarding the most appropriate treatment regimen should be guided by clinical judgment and patient preferences, taking into account drug susceptibility testing (DST) results, prior treatment history, the risk of adverse events, and the severity and site of disease. All treatments should adhere to WHO-recommended standards, which emphasize patient-centered care and support, informed consent where applicable, compliance with good clinical practice, active drug safety monitoring, and regular assessment of treatment effectiveness and the emergence of drug resistance.

#### 4.1.2. Vaccines

In terms of tuberculosis (TB) prevention, the Bacille Calmette–Guérin (BCG) vaccine remains the only licensed vaccine for human use and is widely administered globally [40,41]. First developed in 1921 by Calmette and Guérin at the Pasteur Institute in France, its immunological mechanisms were not well understood at the time [42]. The vaccine was produced using live attenuated strains of *Mycobacterium bovis*—which shares more than 90% genetic similarity with *Mycobacterium tuberculosis*—combined with bovine bile [20]. Today, BCG is a cornerstone of TB prevention strategies, particularly due to its demonstrated efficacy in young children and in preventing severe forms of the disease, such as TB meningitis [40]. It is also considered safe and cost-effective, particularly in low- and middle-income countries. However, the vaccine offers limited protection in adults, underscoring the urgent need for more effective TB vaccines [20].

Given the limited efficacy of the Bacille Calmette–Guérin (BCG) vaccine in adults and the persistent global burden of tuberculosis (TB), multiple next-generation TB vaccines are currently undergoing clinical evaluation. These investigational vaccines aim to provide broader and more durable protection against both drug-sensitive and drug-resistant forms of TB, and some are also being explored for their potential to improve treatment outcomes. As of August 2024, there were 15 vaccine candidates in clinical trials: four in Phase I, five in Phase II, and six in Phase III. These candidates target both the prevention of TB infection and the progression to active disease [43].

Among the most promising candidates, the M72:AS01E vaccine demonstrated an overall efficacy of 54% (95% CI, 2.1–74.2) in preventing pulmonary TB in individuals who received at least one dose [44]. Another candidate, the DAR-901 vaccine—an inactivated whole-cell vaccine—was evaluated in a study involving 667 healthy Tanzanian adolescents for its ability to prevent latent TB infection (LTBI), as measured by interferon-γ release assay (IGRA) conversion. However, it did not demonstrate statistically significant efficacy, with conversion rates of 3% (95% CI, −13.9 to 17.7) for new IGRA positivity and 4% (95% CI, −12.1 to 18.5) for persistent LTBI [45].

Additionally, two live vaccine candidates—MTBVAC and VPM1002—are in development. MTBVAC is derived from a genetically attenuated strain of *Mycobacterium tuberculosis*, in which two virulence genes (*phoP*, a transcriptional regulator, and *fadD26*, involved in lipid metabolism) have been deleted. VPM1002 is a recombinant BCG-based vaccine designed to enhance immunogenicity and safety [43].

Recent innovations in TB vaccine research include mRNA-based platforms, which have entered Phase I clinical trials. These vaccines are being developed for two distinct populations: (i) individuals previously vaccinated with BCG and infected with *M. tuberculosis*, and (ii) individuals with neither prior BCG vaccination nor infection. However, given the novelty of this approach in the TB field, data remain limited and their potential efficacy is yet to be established [40,43].

#### 4.1.3. Social Approaches

Effectively addressing drug-resistant tuberculosis (DR-TB) requires the implementation of comprehensive social interventions that support patient-centered care. Community-based care models, which engage trained community health workers to educate, support, and monitor patients throughout treatment, are particularly effective in improving adherence and outcomes—especially among vulnerable populations such as people living with HIV and migrants. Health education and public awareness campaigns also play a vital role in TB control and should leverage diverse communication platforms, including social media and local outreach events, to disseminate accurate and accessible information about the disease and the importance of completing treatment.

Improving social determinants of health—such as access to nutritious food, stable housing, and quality healthcare—can significantly enhance treatment success rates. Broader efforts to alleviate poverty and expand access to essential services are also critical for reducing transmission in high-risk communities, making these social strategies a cornerstone of effective DR-TB control.

Supportive environments that facilitate access to primary healthcare services encourage timely healthcare-seeking behavior, enabling early detection and prompt initiation of appropriate therapy. Strengthening community health systems and addressing barriers such as stigma, transportation costs, and lack of awareness are essential for ensuring equitable access to care. In settings where these factors are neglected, TB control efforts often fail to reach the most affected populations. Conditions such as poverty, overcrowded housing, malnutrition, and limited healthcare access contribute to the continued spread of TB and hinder adherence to lengthy and complex treatment regimens.

## 5. Conclusions

Despite recent progress in tuberculosis (TB) control, the rise in drug-resistant strains and the impact of the COVID-19 pandemic have posed significant challenges, particularly in diagnosis, treatment adherence, and healthcare accessibility. Strengthening diagnostic capacity with rapid molecular tools, ensuring equitable access to modern therapies, and implementing patient-centered care models are essential steps forward. Public education, antibiotic stewardship, and targeted support for vulnerable populations must also be prioritized. These conclusions further highlight the urgent need for comprehensive and standardized reporting of extensively drug-resistant TB (XDR-TB) cases across EU countries, as improved surveillance would enable a more accurate assessment of resistance patterns and better inform public health strategies. Additionally, sustained investment in vaccine development and international cooperation remain critical to achieving the WHO’s goal of ending TB by 2030.

## Figures and Tables

**Table 1:** Total tuberculosis (TB) reported cases obtained from the European Center for Disease Prevention and Control (ECDC) website. ↑: represents the increase in reported ΤΒ cases in post-pandemic era, ↓: represents the decrease in reported ΤΒ cases in post-pandemic era.

	Pre Pandemic					Pandemic		Post Pandemic
Regions	2015	2016	2017	2018	2019	2020	2021	2022
**Increase in Reported Cases Post Pandemic**								
Cyprus	63	60	53	52	**69**	36	48	96 ↑
Iceland	7	6	14	8	**13**	12	7	17 ↑
Norway	313	295	261	208	**166**	157	155	174 ↑
**Decrease in reported cases post pandemic**								
European Union Total cases	54,721	53,850	50,854	47,683	**45,201**	33,805	33,676	36,179 ↓
Austria	583	634	569	482	**474**	388	396	372 ↓
Belgium	977	1042	967	977	**963**	825	868	852 ↓
Bulgaria	1660	1603	1463	1358	**1344**	930	687	792 ↓
Croatia	488	464	378	372	**305**	198	173	212 ↓
Czechia	517	516	501	443	**461**	363	357	384 ↓
Denmark	357	330	275	291	**284**	221	218	225 ↓
Estonia	217	192	176	147	**150**	124	112	129 ↓
Finland	274	234	244	227	**226**	174	170	190 ↓
France	4744	5735	5006	5048	**5183**	4515	4207	4040 ↓
Germany	5837	5925	5516	5495	**4817**	4186	3939	4076 ↓
Greece	482	440	467	432	**459**	396	206	320 ↓
Hungary	906	786	685	640	**552**	406	335	440 ↓
Ireland	283	315	300	310	**257**	236	217	216 ↓
Italy	3769	4032	3944	3912	**3346**	2287	2480	2439 ↓
Latvia	721	660	552	*	*	*	261	319 ↓
Lithuania	1507	1442	1387	1142	**1058**	726	646	738 ↓
Luxembourg	30	29	32	42	**50**	34	35	48 ↓
Malta	32	50	42	55	**98**	140	54	61 ↓
The Netherlands	862	887	783	795	**754**	622	681	635 ↓
Poland	6430	6444	5787	5487	**5321**	3388	3704	4314 ↓
Portugal	2195	1928	1914	1926	**1907**	1521	1533	1514 ↓
Romania	15,183	13,601	12,997	12,199	**11,618**	7693	7976	9270 ↓
Slovakia	317	296	249	281	**214**	158	137	155 ↓
Slovenia	130	118	112	99	**101**	77	80	74 ↓
Spain	5020	5070	5660	4766	**4532**	3666	3640	3698 ↓
Sweden	815	714	519	488	**479**	324	353	378 ↓
**No differentiation in reported cases post pandemic**								
Liechtenstein	2	2	1	1	*	2	1	1
**Not enough data for comparison**								
UK	6228	6116	5531	5036	5132	*	*	*

*: Represent no data available.

**Table 2:** Multidrug-resistant (MDR) tuberculosis (TB) reported cases obtained from the European Center for Disease Prevention and Control (ECDC) website. ↑: represents the increase in % reported MDR-TB cases in post-pandemic era, ↓: represents the decrease in % reported MDR-TB cases in post-pandemic era.

	Pre Pandemic					Pandemic		Post Pandemic
Regions	2015	2016	2017	2018	2019	2020	2021	2022
**Increase in MDR Cases Post Pandemic (% of Total TB Reported Cases)**								
European Union	4.4	3.9	3.9	3.5	**3.2**	3.3	3.3	4 ↑
Austria	2.8	3.6	4.2	5.2	**1.2**	0.4	3.2	4.4 ↑
Cyprus	0	2.4	2.6	0	**1.9**	0	8.1	2.7 ↑
Czechia	2.4	1.6	2.3	3.7	**2.4**	4.3	3.9	5.6 ↑
Denmark	2.2	1.6	0.9	1.8	**1.8**	1.1	0.6	3.5 ↑
Estonia	21.2	15.8	25.4	24.6	**20.5**	18.1	28.6	23.7 ↑
Finland	3.8	3.3	2.6	2.2	**1.7**	0.7	2.5	5.6 ↑
Germany	2.8	2.6	3.1	2.9	**2.5**	2.7	2.5	5.5 ↑
Greece	4.3	3.4	3.7	3.2	**2.7**	2.3	6.1	3.7 ↑
Hungary	2.1	5.3	3.5	3.6	**4.6**	4	6.1	6.4 ↑
Iceland	0	0	0	0	**0**	0	0	7.7 ↑
Ireland	0.5	2.6	2.5	2.4	**1.7**	1	5.7	9.9 ↑
Italy	2.7	2.6	2.5	2.1	**2.2**	2.9	2.8	2.8 ↑
Latvia	11.1	10.4	10.6	*	*	*	8.8	10.9 ↑
Luxembourg	0	4	4.3	2.9	**3.1**	0	0	3.2 ↑
Malta	4.5	0	0	0	**0**	0	0	2.6 ↑
The Netherlands	1.4	2.5	1.7	1.1	**1.4**	2.4	1.9	2 ↑
Norway	2	4.8	4.2	2.4	**1.5**	0.8	7.3	7.2 ↑
Poland	0.8	1.1	1.2	1.3	**1.1**	1.6	1.9	3.1 ↑
Portugal	1.8	1.3	0.9	0.9	**1**	2.1	1.3	1.5 ↑
Slovakia	1.3	2.7	4.5	1.4	**4.4**	1.5	5.3	6.7 ↑
Slovenia	0	0	0.9	0	**0**	0	1.3	1.5 ↑
Sweden	3.2	3.7	2.6	3.2	**1.8**	2.3	2.1	4.7 ↑
**Decrease in MDR cases post pandemic (% of total TB reported cases)**								
Belgium	2	1.3	0.7	1	**2.1**	2.3	1.3	1.4 ↓
Bulgaria	3.6	2.8	4	4.4	**2.4**	3.7	2.3	2.2 ↓
Croatia	0.3	0	0	0.7	**0.4**	0.6	0	0 ↓
Lithuania	19.7	20.3	21.7	17.1	**17**	16.2	17	16.5 ↓
Romania	6	5.5	4.5	4.4	**4**	4.3	3.5	3.3 ↓
Spain	4.8	4.4	1.7	1.8	**1.2**	1.5	1.6	1 ↓
**Not enough data for comparison**								
France	*	1.7	*	*	*	*	*	*
Liechtenstein	0	0	0	0	*	*	0	*
UK	1.3	1.5	1.4	1.3	1.2	*	*	*

*: Represent no data available.

**Table 3:** Rifampicin-resistant/multidrug-resistant (RR/MDR) tuberculosis (TB) reported cases obtained from the European Center for Disease Prevention and Control (ECDC) website. ↑: represents the increase in % reported RR/MD-TB cases in post-pandemic era, ↓: represents the decrease in % reported RR/MDR-TB cases in post-pandemic era.

	Pre Pandemic					Pandemic		Post Pandemic
Regions	2015	2016	2017	2018	2019	2020	2021	2022
**Increase in RR/MDR Cases Post Pandemic (% of Total TB Reported Cases)**								
European Union	4.6	4.8	4.6	4.6	**3.7**	3.9	3.9	4.6 ↑
Austria	4.4	3.6	4.5	4.4	**1.8**	0.7	3.2	4.4 ↑
Croatia	1.2	0	0	1.2	**0.4**	0.6	0	1.2 ↑
Cyprus	3.9	2.4	2.6	3.9	**1.9**	0	8.1	3.9 ↑
Czechia	5.5	1.9	2.3	5.5	**2.4**	4.3	3.9	5.5 ↑
Denmark	4.1	2	0.9	4.1	**1.7**	1.1	0.6	4.1 ↑
Estonia	23.6	16.3	25.2	23.6	**21.6**	18.9	28.9	23.6 ↑
Finland	5.6	3.3	3.1	5.6	**1.7**	0.7	2.5	5.6 ↑
Germany	6.3	2.9	3.5	6.3	**2.8**	3.3	3.4	6.3 ↑
Greece	5.2	4.2	4.5	5.2	**3.1**	3.4	6	5.2 ↑
Hungary	6.3	5.8	4.1	6.3	**4.8**	6.2	7.2	6.3 ↑
Iceland	7.7	0	0	7.7	**0**	0	0	7.7 ↑
Ireland	7.9	3	3	7.9	**3.3**	0.8	6.3	7.9 ↑
Italy	3.5	*	3.7	3.5	**2.6**	3.3	3.1	3.5 ↑
Luxembourg	3.2	4	4.3	3.2	**3.1**	0	0	3.2 ↑
Malta	5.3	0	0	5.3	**0**	3.8	2.7	5.3 ↑
The Netherlands	2	2.7	2.1	2	**1.9**	3.1	2.3	2 ↑
Norway	7.3	4.7	4	7.3	**1.5**	1.4	9.2	7.3 ↑
Poland	3.3	1.2	1.4	3.3	**1.2**	1.7	2	3.3 ↑
Slovakia	6.7	2.7	5.2	6.7	**4.4**	1.5	6.7	6.7 ↑
Slovenia	1.5	0	0.9	1.5	**0**	1.4	1.3	1.5 ↑
Spain	2	1.5	2.1	2	**1.5**	2.4	2.2	2 ↑
Sweden	5	4.2	2.8	5	**2.5**	2.3	2.4	5 ↑
**Decrease in RR/MDR cases post pandemic (% of total TB reported cases)**								
Bulgaria	2.2	3.3	4.7	2.2	**3**	4.8	2.3	2.2 ↓
Lithuania	17.3	21.2	23	17.3	**18.5**	16.5	18.2	17.3 ↓
Romania	4	6.4	5.5	4	**4.9**	5.1	4.5	4 ↓
**No differentiation in RR/MDR cases post pandemic (% of total TB reported cases)**								
Belgium	2.4	1.6	1.1	2.4	**2.4**	2.8	1.3	2.4
Latvia	12.1	10.4	11.1	12.1	*	*	9.8	12.1
Portugal	1.7	1.7	1.2	1.7	**1.7**	2.4	1.5	1.7
**Not enough data for comparison**								
France	*	*	*	*	*	*	*	*
Liechtenstein	*	0	0	*	*	*	0	*
UK	*	1.7	1.8	*	1.7	*	*	*

*: Represent no data available.

**Table 4:** The top eight EU countries showing increased proportions of MDR and RR/MDR tuberculosis cases between 2019 (pre-pandemic) and 2022 (post-pandemic).

EU Countries	% MDR Cases’ Increase Rate (Comparison 2019 vs. 2022)	% RR/MDR Cases’ Increase Rate (Comparison 2019 vs. 2022)
Austria	+266.67%	+144.44%
Czechia	+133.33%	+129.2%
Finland	+229.41%	+229.41%
Germany	+120%	+125%
Ireland	+482.35%	+139.39%
Norway	+380%	+386.67%
Poland	+181.8%	+175%
Sweden	+161.11%	+100%
**Average**	**244.33%**	**178.64%**

**Table 5:** The five EU countries showing a great divergence between the % MDR and RR/MDR cases between 2019 (pre-pandemic data) and 2022 (post-pandemic data). Blue font: % decrease between 2019 and 2022 data; red font: % increase between 2019 and 2022 data; Black font: no difference between 2019 and 2022 data. ↑: represents the increase in % reported MDR or in RR/MDR TB cases in post-pandemic era, ↓: represents the decrease in % reported MDR or in RR/MDR TB cases in post-pandemic era.

EU Countries	% MDR Cases in Post-Pandemic Era (2022 Data)	% RR/MDR Cases in Post-Pandemic Era (2022 Data)
Belgium	1.4 ↓	2.4
Croatia	0 ↓	1.2 ↑
Latvia	10.9 ↑	12.1
Portugal	1.5 ↑	1.7
Spain	1 ↓	2 ↑

**Table 6:** Treatment success rates of multidrug-resistant MDR tuberculosis (TB) reported cases for a 24-month therapy obtained from the European Center for Disease Prevention and Control (ECDC) website. ↑: represents the increase in % treatment success rates in MDR-TB cases in post-pandemic era, ↓: represents the decrease in % treatment success rates in MDR-TB cases in post-pandemic era.

	Pre Pandemic					Pandemic		Post Pandemic
Regions	2015	2016	2017	2018	2019	2020	2021	2022
**Increase in % Treatment Success Rates of MDR-TB Cases During the Pandemic (24-Month Regimen)**								
European Union	43.9	44.2	44.3	50.8	**47.9**	48.6 ↑	*	*
Estonia	52.6	70.8	75	73.3	**73.1**	73.7 ↑	*	*
Hungary	25	44.4	45.5	41.7	**25**	42.9 ↑	*	*
Lithuania	38.6	42.2	41.4	52.4	**42.8**	50.5 ↑	*	*
The Netherlands	50	71.4	66.7	83.3	**85.7**	100 ↑	*	*
Norway	100	90.9	88.9	100	**50**	100 ↑	*	*
Portugal	30.4	46.7	70	60	**33.3**	85.7 ↑	*	*
Slovakia	0	0	16.7	50	**80**	100 ↑	*	*
**Decrease in % treatment success rates of MDR-TB cases during the pandemic (24-month regimen)**								
Austria	91.7	64.7	50	83.3	**100**	0 ↓	*	*
Belgium	100	90	80	85.7	**86.7**	64.3 ↓	*	*
Czechia	33.3	16.7	37.5	33.3	**50**	9.1 ↓	*	*
Germany	39.3	33.7	52.5	60.7	**43.7**	41.7 ↓	*	*
Romania	41.3	47.4	36	39.5	**45.8**	43.1 ↓	*	*
Sweden	90.9	90.9	81.8	100	**100**	83.3 ↓	*	*
**No differentiation in % treatment success rates of MDR-TB cases during the pandemic** **(24-month regimen)**								
Croatia	100	*	*	*	*	100	*	*
**Not enough data for comparison**								
Bulgaria	58.3	52.6	66.7	62.5	*	*	*	*
Cyprus	*	*	*	*	*	*	*	*
Denmark	0	75	50	*	*	*	*	*
Finland	37.5	66.7	20	50	66.7	*	*	*
France	*	*	*	*	*	*	*	*
Greece	*	*	*	*	*	*	*	*
Iceland	*	*	*	*	*	*	*	*
Ireland	*	83.3	40	*	33.3	*	*	*
Italy	*	*	*	*	*	*	*	*
Latvia	61.9	0	*	*	*	*	*	*
Liechtenstein	*	*	*	*	*	*	*	*
Luxembourg	*	*	*	*	*	*	*	*
Malta	*	*	*	*	*	*	*	*
Poland	31.4	17.4	*	*	*	*	*	*
Slovenia	*	*	0	*	*	*	*	*
Spain	*	*	*	*	*	*	*	*
UK	62.5	58.6	58	*	*	*	*	*

*: Represent no data available.

**Table 7:** Treatment success rates of multidrug-resistant MDR tuberculosis (TB) reported cases for a 12-month therapy obtained from the European Center for Disease Prevention and Control (ECDC) website. ↑: represents the increase in % treatment success rates in MDR-TB cases in post-pandemic era, ↓: represents the decrease in % treatment success rates in MDR-TB cases in post-pandemic era.

	Pre Pandemic					Pandemic		Post Pandemic
Regions	2015	2016	2017	2018	2019	2020	2021	2022
**Increase in % Treatment Success Rates of MDR-TB Cases During the Pandemic (12-Month Regimen)**								
European Union	62.9	68.9	70.7	67.7	**65.4**	62.7	62.9 ↑	*
Cyprus	55.6	65	66	59.6	**26.1**	47.2	50 ↑	*
Estonia	69.1	69.8	64.2	66	**63.3**	64.5	73.2 ↑	*
Slovenia	81.5	71.2	75.9	77.8	**70.3**	74	73.8 ↑	*
Spain	71.4	70.4	62.8	44.8	**47.2**	54.8	49.7 ↑	*
Sweden	84.4	88.2	84.6	76.2	**64.5**	74.1	85 ↑	*
**Decrease in % treatment success rates of MDR-TB cases during the pandemic (12-month regimen)**								
Belgium	78	81	78.9	80.9	**79.5**	67.3	77 ↓	*
Bulgaria	83.4	83.9	83	84.5	**81**	*	78.6 ↓	*
Croatia	57	58	65.1	64.5	**60**	60.1	46.8 ↓	*
Czechia	67.7	67.4	68.1	66.6	**72**	67.5	65.8 ↓	*
Denmark	47.9	40.6	40	17.5	**44**	34.4	19.3 ↓	*
Finland	37.2	30.8	36.1	22.9	**38.1**	25.3	6.5 ↓	*
France	53.4	45.8	48.7	41.2	**36.9**	36.4	36.4 ↓	*
Germany	63.7	68.3	69.3	77.2	**66.8**	63.6	65.5 ↓	*
Hungary	69.6	69.2	65.7	63.9	**62.9**	66	62.4 ↓	*
Iceland	100	83.3	92.9	100	**100**	33.3	57.1 ↓	*
Ireland	70	56.8	58	37.1	**22.2**	6.4	3.7 ↓	*
Lithuania	66	68.1	67.1	72.3	**72.8**	73.3	71.7 ↓	*
The Netherlands	86.1	86.2	86.2	85.8	**85.4**	82.5	80.3 ↓	*
Norway	85.6	83.1	89.3	86.1	**84.3**	87.3	81.9 ↓	*
Portugal	71.9	71.6	72.5	72.5	**74.1**	72.3	67.1 ↓	*
Romania	80.2	81.3	81.8	79.8	**79.4**	77.1	77.2 ↓	*
Slovakia	85.5	85.1	88	90.7	**91.6**	83.5	86.1 ↓	*
**No differentiation in % treatment success rates of MDR-TB cases during the pandemic** **(12-month regimen)**								
Austria	71.7	72.7	66.8	70.7	73.2	69.1	73.2	*
Liechtenstein	50	100	100	*	*	100	100	*
**Not enough data for comparison**								
Greece	*	*	*	*	*	*	*	*
Italy	*	*	*	*	*	*	*	*
Latvia	76.4	77.6	*	*	*	*	*	*
Luxembourg	*	*	0	*	38	38.2	.	*
Malta	*	*	*	*	*	99.3	98.1	*
Poland	52.7	53.3	*	*	*	*	*	*
UK	79.4	80.6	81.1	78.3	*	*	*	*

*: Represent no data available.

**Table 8:** The four EU countries showing simultaneous decline in both 24-month and 12-month therapy success rates between 2019 (pre-pandemic data) and 2020/2021 (during COVID-19 pandemic).

EU Countries	% Decrease Success Rate in 24-Month Therapy (Comparison 2019 vs. 2020)	% Decrease Success Rate in 12-Month Therapy (Comparison 2019 vs. 2021)
Belgium	−25.84%	−3.14%
Czechia	−81.8%	−8.61%
Germany	−4.58%	−1.98%
Romania	−5.90%	−2.77%

## Data Availability

All data used for the meta-analysis are publicly available on the European Center for Disease Prevention and Control (ECDC) website: https://atlas.ecdc.europa.eu/public/index.aspx (accessed on 1 July 2025).

## Supplementary Materials

The following supporting information can be downloaded at: https://www.mdpi.com/article/10.3390/ph18101535/s1, completed PRISMA checklist for systematic reviews. References [46,47] are cited in the Supplementary Materials.

## Abbreviations

The following abbreviations are used in this manuscript:

AMR	Antimicrobial resistance
BCG	Bacille Calmette-Guérin
CI	Contact investigation
DOT	Directly Observed Therapy
DST	Drug Susceptibility Testing
DS-TB	Drug-Sensitive Tuberculosis
ECDC	European Centre for Disease Prevention and Control
EU/EEA	European Union/European Economic Area
LTBI	Latent Tuberculosis Infection
MDR-TB	Multidrug-Resistant Tuberculosis
MTB/RIF	Mycobacterium tuberculosis/Rifampicin
MTBC	Mycobacterium Tuberculosis Complex
NTM	Nontuberculous mycobacteria
Pre-XDR-TB	Pre-extensively drug-resistant tuberculosis
RR/MDR-TB	Rifampicin-resistant/Multidrug-resistant tuberculosis
RR-TB	Rifampicin-resistant tuberculosis
TB	Tuberculosis
WGS	Whole-Genome Sequencing
XDR-TB	Extensively drug-resistant tuberculosis
