# Supplementary material for: Impact of the COVID-19 Pandemic on Drug-Resistant Tuberculosis in Europe: A Meta-Analysis of Epidemiological Trends
Source: Pharmaceuticals (Basel). 2025 Oct 12;18(10):1535. doi: 10.3390/ph18101535 (PMC12566819; doi:10.3390/ph18101535)
Supplement: Supplementary file 1 [file pharmaceuticals-18-01535-s001.zip › pharmaceuticals-3822584-supplementary.pdf]

PRISMA 2020 Checklist

Section and Topic	Item #	Checklist item	Location where item is reported
**TITLE**			
Title	1	Identify the report as a systematic review.	Title identifies manuscript as a systematic review (p.1)
**ABSTRACT**			
Abstract	2	See the PRISMA 2020 for Abstracts checklist.	Abstract includes objectives, data source (ECDC), methods (meta-analysis of epidemiological trends), results and conclusions. (p.1)
**INTRODUCTION**			
Rationale	3	Describe the rationale for the review in the context of existing knowledge.	Provided: COVID-19 disrupted TB surveillance/treatment, raising concern for drug resistance. (p. 2-3)
Objectives	4	Provide an explicit statement of the objective(s) or question(s) the review addresses.	Aim stated: assess epidemiological trends of drug-resistant TB across EU before, during, after pandemic. (p. 3)
**METHODS**			
Eligibility criteria	5	Specify the inclusion and exclusion criteria for the review and how studies were grouped for the syntheses.	Data from 31 EU countries reported to ECDC (2015–2022). No additional eligibility restrictions. (p.3-4, lines 140-150)
Information sources	6	Specify all databases, registers, websites, organisations, reference lists and other sources searched or consulted to identify studies. Specify the date when each source was last searched or consulted.	ECDC official database (2015–2022) https://atlas.ecdc.europa.eu/public/index.aspx, accessed in 28 July 2025
Search strategy	7	Present the full search strategies for all databases, registers and websites, including any filters and limits used.	Not applicable – single official database (ECDC). No literature search performed.
Selection process	8	Specify the methods used to decide whether a study met the inclusion criteria of the review, including how many reviewers screened each record and each report retrieved, whether they worked independently, and if applicable, details of automation tools used in the process.	Not applicable – no screening of individual studies; all available ECDC data included.
Data collection process	9	Specify the methods used to collect data from reports, including how many reviewers collected data from each report, whether they worked independently, any processes for obtaining or confirming data from study investigators, and if applicable, details of automation tools used in the process.	Data extracted directly from ECDC official reports and surveillance databases by study team.
Data items	10a	List and define all outcomes for which data were sought. Specify whether all results that were compatible with each outcome domain in each study were sought (e.g. for all measures, time points, analyses), and if not, the methods used to decide which results to collect.	TB incidence cases, MDR-TB, RR/MDR-TB cases, treatment success rates (12-/24-month).
	10b	List and define all other variables for which data were sought (e.g. participant and intervention characteristics, funding sources). Describe any assumptions made about any missing or unclear information.	TB incidence cases, MDR-TB, RR/MDR-TB cases, treatment success rates (12-/24-month).
Study risk of bias assessment	11	Specify the methods used to assess risk of bias in the included studies, including details of the tool(s) used, how many reviewers assessed each study and whether they worked independently, and if applicable, details of automation tools used in the process.	Not applicable – analysis based on official surveillance data, not individual studies.
Effect measures	12	Specify for each outcome the effect measure(s) (e.g. risk ratio, mean difference) used in the synthesis or presentation of results.	Changes in TB incidence cases and proportions of MDR/RR-MDR cases across timeframes; treatment success rates.
Synthesis methods	13a	Describe the processes used to decide which studies were eligible for each synthesis (e.g. tabulating the study intervention characteristics and comparing against the planned groups for each synthesis (item #5)).	Meta-analysis of pooled ECDC data by year and country, stratified by pre-/during-/post-pandemic periods. (p.3-4)

Section and Topic	Item #	Checklist item	Location where item is reported
	13b	Describe any methods required to prepare the data for presentation or synthesis, such as handling of missing summary statistics, or data conversions.	Meta-analysis of pooled ECDC data by year and country, stratified by pre-/during-/post-pandemic periods. (p.3-4)
	13c	Describe any methods used to tabulate or visually display results of individual studies and syntheses.	Meta-analysis of pooled ECDC data by year and country, stratified by pre-/during-/post-pandemic periods. (p.3-4)
	13d	Describe any methods used to synthesize results and provide a rationale for the choice(s). If meta-analysis was performed, describe the model(s), method(s) to identify the presence and extent of statistical heterogeneity, and software package(s) used.	Meta-analysis of pooled ECDC data by year and country, stratified by pre-/during-/post-pandemic periods. (p.3-4)
	13e	Describe any methods used to explore possible causes of heterogeneity among study results (e.g. subgroup analysis, meta-regression).	Meta-analysis of pooled ECDC data by year and country, stratified by pre-/during-/post-pandemic periods. (p.3-4)
	13f	Describe any sensitivity analyses conducted to assess robustness of the synthesized results.	Meta-analysis of pooled ECDC data by year and country, stratified by pre-/during-/post-pandemic periods. (p.3-4)
Reporting bias assessment	14	Describe any methods used to assess risk of bias due to missing results in a synthesis (arising from reporting biases).	Not applicable – no study selection; all official ECDC data from 2015 to 2022 were used.
Certainty assessment	15	Describe any methods used to assess certainty (or confidence) in the body of evidence for an outcome.	Not formally conducted; limitations acknowledged (e.g., missing XDR-TB data, reporting heterogeneity).
**RESULTS**			
Study selection	16a	Describe the results of the search and selection process, from the number of records identified in the search to the number of studies included in the review, ideally using a flow diagram.	Not applicable – no literature screening.
	16b	Cite studies that might appear to meet the inclusion criteria, but which were excluded, and explain why they were excluded.	Not applicable – no literature screening.
Study characteristics	17	Cite each included study and present its characteristics.	Surveillance data from 31 EU countries, 2015–2022. Official data from ECDC database
Risk of bias in studies	18	Present assessments of risk of bias for each included study.	Not applicable.
Results of individual studies	19	For all outcomes, present, for each study: (a) summary statistics for each group (where appropriate) and (b) an effect estimate and its precision (e.g. confidence/credible interval), ideally using structured tables or plots.	Tables and graphical plots are presented in the manuscript to illustrate, for each country, annual trends in TB cases, MDR and RR/MDR-TB proportions, and treatment outcomes between 2015 and 2022 (p. 5-17)
Results of syntheses	20a	For each synthesis, briefly summarise the characteristics and risk of bias among contributing studies.	Not applicable.
	20b	Present results of all statistical syntheses conducted. If meta-analysis was done, present for each the summary estimate and its precision (e.g. confidence/credible interval) and measures of statistical heterogeneity. If comparing groups, describe the direction of the effect.	Reported pooled trends across pre-/during-/post-pandemic periods (decline in TB cases, rise in MDR/RR-MDR proportion, treatment outcomes). (p. 5-17)
	20c	Present results of all investigations of possible causes of heterogeneity among study results.	Not applicable.
	20d	Present results of all sensitivity analyses conducted to assess the robustness of the synthesized results.	Not applicable.
Reporting biases	21	Present assessments of risk of bias due to missing results (arising from reporting biases) for each synthesis assessed.	Not applicable.

PRISMA 2020 Checklist

Section and Topic	Item #	Checklist item	Location where item is reported
Certainty of evidence	22	Present assessments of certainty (or confidence) in the body of evidence for each outcome assessed.	The analysis was based on official ECDC data, with minor limitations from incomplete country reporting and lack of XDR-TB case data. (p.17)
**DISCUSSION**			
Discussion	23a	Provide a general interpretation of the results in the context of other evidence.	COVID-19 pandemic correlates with disruption of TB control, rising MDR burden, and the challenges in treatment outcomes. (p. 23-24)
	23b	Discuss any limitations of the evidence included in the review.	XDR-TB data missing, reliance on reported surveillance data with date gaps in some countries, potential underdiagnosis due to the pandemic. (p.17)
	23c	Discuss any limitations of the review processes used.	Not applicable.
	23d	Discuss implications of the results for practice, policy, and future research.	Not applicable.
**OTHER INFORMATION**			
Registration and protocol	24a	Provide registration information for the review, including register name and registration number, or state that the review was not registered.	PROSPERO (registration number: CRD420251153770)
	24b	Indicate where the review protocol can be accessed, or state that a protocol was not prepared.	https://www.crd.york.ac.uk/PROSPERO/view/CRD420251153770
	24c	Describe and explain any amendments to information provided at registration or in the protocol.	Not applicable
Support	25	Describe sources of financial or non-financial support for the review, and the role of the funders or sponsors in the review.	This research received no external funding
Competing interests	26	Declare any competing interests of review authors.	The authors declare no conflicts of interest.
Availability of data, code and other materials	27	Report which of the following are publicly available and where they can be found: template data collection forms; data extracted from included studies; data used for all analyses; analytic code; any other materials used in the review.	ECDC official database: https://atlas.ecdc.europa.eu/public/index.aspx

From: Page MJ, McKenzie JE, Bossuyt PM, Boutron I, Hoffmann TC, Mulrow CD, et al. The PRISMA 2020 statement: an updated guideline for reporting systematic reviews. BMJ 2021;372:n71. doi: 10.1136/bmj.n71. This work is licensed under CC BY 4.0. To view a copy of this license, visit https://creativecommons.org/licenses/by/4.0/
